# YAHA: fast and flexible long-read alignment with optimal breakpoint detection

**DOI:** 10.1093/bioinformatics/bts456

**Published:** 2012-07-24

**Authors:** Gregory G. Faust, Ira M. Hall

**Affiliations:** ^1^Department of Computer Science, ^2^Department of Biochemistry and Molecular Genetics and ^3^Center for Public Health Genomics, University of Virginia, Charlottesville, 22908 VA, USA

## Abstract

**Motivation:** With improved short-read assembly algorithms and the recent development of long-read sequencers, split mapping will soon be the preferred method for structural variant (SV) detection. Yet, current alignment tools are not well suited for this.

**Results:** We present YAHA, a fast and flexible hash-based aligner. YAHA is as fast and accurate as BWA-SW at finding the single best alignment per query and is dramatically faster and more sensitive than both SSAHA2 and MegaBLAST at finding all possible alignments. Unlike other aligners that report all, or one, alignment per query, or that use simple heuristics to select alignments, YAHA uses a directed acyclic graph to find the optimal set of alignments that cover a query using a biologically relevant breakpoint penalty. YAHA can also report multiple mappings per defined segment of the query. We show that YAHA detects more breakpoints in less time than BWA-SW across all SV classes, and especially excels at complex SVs comprising multiple breakpoints.

**Availability:** YAHA is currently supported on 64-bit Linux systems. Binaries and sample data are freely available for download from http://faculty.virginia.edu/irahall/YAHA.

**Contact:** imh4y@virginia.edu

## 1 INTRODUCTION

Structural variation (SV) is a major source of diversity in germline and cancer genomes, but is difficult to map relative to other forms of variation. Since 2008, most sequence-based studies of SV have used paired-end mapping (PEM), which relies upon clustering of discordant paired-end reads that map to either side of an SV breakpoint. Now, with the rapid improvement of short-read assembly algorithms and the development of third-generation long-read sequencing technologies, split-read or split-contig mapping (we refer to both as SRM) will soon be the preferred method. SRM is significantly more precise and less error prone than PEM. Yet, current read mappers are not well designed for aligning breakpoint-containing query sequences. Here, we present YAHA, a flexible hash-based aligner that is explicitly designed for optimal SV breakpoint detection from long query sequences.

To accurately determine SV breakpoints using SRM, an aligner must do four things well. First, it must accurately determine the best set of alignments that cover the length of the query; the ‘Optimal Coverage Set’ (OCS). This is best accomplished by using an algorithm that provides provably optimal results given some objective function. Our use of a best-path algorithm on a directed acyclic graph (DAG) of alignments does just that. The objective function is specifically tuned to finding SV events by taking into account the length and quality of alignments, the number of alignments in the OCS and the genomic distance between those alignments. Second, it must be able to report alignments similar to those in the OCS in order to allow for the use of combinatorial breakpoint detection algorithms that cluster multiple mappings per read (Hormozdiari *et al.*, 2009; Quinlan *et al.*, 2010). YAHA’s use of an optimal DAG algorithm for discovery of the OCS and its ability to find collections of alignments similar to the OCS are completely novel. Third, it must be able to generate a large number of viable alignments to feed the above two algorithms. Long-read aligners such as BWA-SW (Li and Durbin, 2010) and AGILE (Misra *et al.*, 2011) severely restrict the number of alignments under consideration early in query processing. While this improves speed, it reduces the likelihood of finding the OCS and precludes finding alignments similar to them. YAHA can produce the required large number of alignments. Optionally, the user can choose to output all of them. Other aligners such as MegaBLAST (Altschul *et al.*, 1990) and SSAHA2 (Ning *et al.*, 2001) can also produce numerous alignments, but have no notion of an OCS. Fourth, the aligner must be able to run in a reasonable amount of time. YAHA uses a unique combination of heuristics and optimizations to accomplish this. We use a hashing scheme similar to SSAHA, but with a considerably faster approach for sorting hash table seeds. We use banded Smith–Waterman (SW) and a modified version of MegaBLAST’s X-Dropoff heuristic for extensions. Finally, we calculate the OCS without unduly impacting performance by using a time and space optimized DAG algorithm. YAHA is the only aligner that does all four of these things well, and therefore is uniquely well suited to SV breakpoint detection. In addition, it is important to score alignments using a metric that is capable of accommodating a wide range of error profiles in order to perform well on queries from diverse sources, including existing (Eid *et al.*, 2009) and future (Schadt *et al.*, 2010) long-read sequencing technologies. To accomplish this, YAHA utilizes Affine Gap Scoring (AGS) with user specified cost/reward parameters.

In the next section, we explain YAHA’s algorithms in more detail. In Section 3, we show the efficacy of these algorithms in three comparison tests. First, we compare YAHA with MegaBLAST and SSAHA2 in their capacity to generate a large number of accurate alignments in a reasonable amount of CPU time. Second, we compare YAHA with BWA-SW in their ability to find the single best alignment over a range of read lengths and error rates. Third, we compare YAHA with BWA-SW in their ability to accurately identify SV breakpoints over a range of SV event categories.

## 2 METHODS

YAHA uses a ‘seed and extend’ strategy for DNA alignment. Alignments are output in SAM format (Li *et al.*, 2009). YAHA breaks the alignment process into six stages. Steps 5, ‘Optimal Query Coverage’ (OQC) and 6, ‘Filter By Similarity’ (FBS), are not included in any other DNA aligner. Although many of the basic algorithms used by YAHA are not novel, their inclusion in a DNA alignment tool is.

### 2.1 Find seed matches

A base-pair sequence of fixed-length *k* is called a ‘*k*-mer’. YAHA uses a hash table index to locate the set of locations (seeds) where each *k*-mer in the query sequence appears as a subsequence of the reference. There are three parameters that control the creation of the index; seed length (*k*), the ‘skip-distance’ between the starting locations of seeds in the reference, and the maximum allowed hits for a *k*-mer before it is considered too repetitive to be useful (‘maxHits’). Typical values of *k* range from 8 to 15. The skip-distance can range from 1 (max overlap) to *k* (no overlap). YAHA builds an index once per desired combination of reference genome and index parameters and stores it in a file. While performing alignments, the index file is accessed via memory-mapped IO as if it were stored in RAM.

**Fig. 1:**
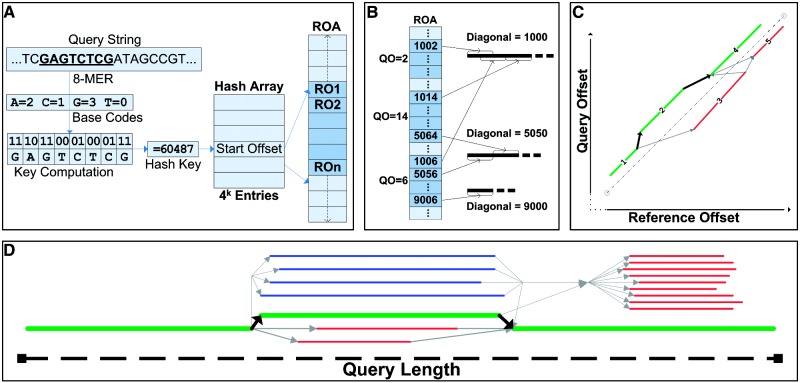
(A) Starting at each location in the query, we form a *k*-mer which is then converted to a hash key by compressing the bases in the *k*-mer using 2-bits per base. That hash key is then used to directly index into the Hash Array, giving the starting offset and length of the subset of the ROA that contains the collection of reference locations for that *k*-mer. (B) Next, seed matches from the query and reference that fall along the same diagonal are collected into extended seeds called ‘fragments’ by merging the pre-sorted ROA regions for each query location using a Binary Heap. (C) In any given region of the reference, many fragments can be included in a potential alignment. YAHA uses a graph algorithm to find the set that maximizes the estimated score. In this example, fragments 1, 2 and 4 form the best alignment. (D) During the Optimal Query Coverage algorithm, we will find the best collection of ‘primary’ alignments (green lines) that has the highest non-overlapping sum of scores. Filter By Similarity is then used to determine the remaining ‘secondary’ alignments (blue lines) that are highly similar to any primary alignment. The remaining alignments (red lines) are not included in the output for the query.

For mammalian genomes, and *k* ≤ 15, a very large percentage of all unique *k*-mers will appear at least once in the genome. Therefore, a natural way to form a hash key is to compress the *k*-mer using 2 bits per base, then use it as an offset into a table with an entry for each *k*-mer (Fig. 1A). The list of reference hits for all keys are concatenated in one large array called the Reference Offset Array (ROA). This index structure is the one used in YAHA and was taken directly from SSAHA. Similar indexing strategies are used by MegaBLAST, BLAT (Kent, 2002), and others. For mammalian genomes, we find that *k* = 15 and skip-distance = 1 (a ‘15/1’ index) performs quite well, and we use such an index for all YAHA test runs discussed below. Like MOSAIK (http://bioinformatics.bc.edu/marthlab/Mosaik), YAHA allows for sampling of hits in the reference down to the specified maxHits parameter setting. This can have a dramatic impact on the trade-off between sensitivity and run-time, as we later show in Section 3.

### 2.2 Combine seed matches into ‘fragments’

Next, seeds are joined together to form extended seeds or ‘fragments’ of contiguous matching bases between the query and the reference (Fig. 1B). Seeds that can be strung together in this way appear on the same ‘diagonal’ in a plot of Reference Offset (RO) versus Query Offset (QO) (Fig. 1C). The query length (QL) determines the number of *k*-mers that appear in the query. Let N equal the sum over QL of the number of reference hits for the *k*-mer starting at each query offset. To find extended seeds, many aligners collect all N seeds for a query into an array and use an *O*(NlogN) sort to collocate seeds to be placed in a fragment. However, since the seeds for each *k*-mer are presorted by RO in the ROA, YAHA instead performs a QL-way merge of the presorted ROA regions. A Priority Queue (Binary Heap) is used to aid the merging process. This approach reduces the complexity to an upper bound of NlogQL because the heap will contain a maximum of QL entries and will become smaller over time as loci on the query exhaust their lists of seed matches. In addition, we do not need to create the large N element array, saving memory footprint and possibly improving cache locality. To the best of our knowledge, no other hash based aligner uses this optimization to reduce the cost of sorting the seed matches.

### 2.3 Combine fragments into an alignment

YAHA next finds the best potential alignment in each region of the reference by combining the fragments that contribute to the highest estimated alignment score in that region. Selecting fragments can be difficult in regions with tandem repeats as there may be numerous overlapping fragments with various distances between their diagonals. We calculate the estimated score for each possible collection of fragments in the region using the AGS parameters; fragments are scored as matches, while differences between fragment diagonals are scored as a single indel. YAHA uses a graph algorithm that finds the path with the maximum estimated score (Fig. 1C). The nodes of the graph (colored lines) represent fragments, and the edges (gray lines) represent the cost or benefit of one fragment succeeding another in the alignment. Since fragments earlier in the query can only be succeeded by fragments later in the query, the graph is directed and acyclic (a ‘DAG’), with a maximum of *n*^2^/2 edges for n fragments. In DAGs, such min/max path algorithms need visit each edge only once in the proper (topological sort) order. By placing the nodes in an array and presorting them by starting QO using a conventional *O*(NlogN) sort, we perform the graph algorithm without ever forming the edges. This saves space and improves cache behavior. Each node is visited sequentially while checking against all nodes above it in the sorted array. If an edge is allowed between two nodes, we immediately score and relax the edge, resetting the best score and best-path back-pointer in the later node when appropriate.

Traditionally, the task of selecting the best seed matches to include in an alignment has been performed by using Dynamic Programming (DP) (Pearson and Lipman, 1988). Straightforward DP implementations require time and space proportional to *n*^2^. However, the Hirschberg algorithm (Myers and Miller, 1988), reduces the DP space requirement to *O*(n), but approximately doubles the runtime. The graph algorithm used in YAHA also uses time proportional to *n*^2^, and space proportional to *n*, but without this added complexity. We reuse this graph algorithm in the OQC phase described below.

Once a best set of fragments is found for a reference region, it is placed into a potential alignment to be completed as described below. If any of the remaining fragments from this reference region do not overlap on the query with any potential alignments already found in this region, they are used in another run of the graph algorithm. This process continues until there are no remaining fragments in an uncovered portion of the query. We next discard all potential alignments that contain a number of seed matches that falls below a user specified threshold (minMatch). It is common for aligners to define this threshold in terms of the number of seed hits from the index. As the seeds can be overlapping, YAHA instead uses a threshold for the total number of non-overlapping bases that appear in seeds. We believe such a threshold is both more accurate and easier for the user to manage.

### 2.4 Complete alignments using DP

YAHA now takes each potential alignment from above, and completes the calculation of the full alignment. It uses a modified version of SW only to find the portions of the alignment that fall between fragments, and to find the best forward and backward extensions for the alignment. Our implementation of SW calculates AGS with a well known strategy to reduce memory usage first proposed by (Gotoh, 1982). YAHA also uses a common heuristic called ‘banding’ that reduces costs by calculating only the DP values near the diagonal of the array. Because the endpoints of extensions are not known, we heuristically use twice the bandwidth during extension as used between fragments, and a simplified version of the well known ‘X-Dropoff’ heuristic (Zhang *et al.*, 1998) which stops extending an alignment when the score for the current extension is more than X below the best score for a shorter extension. YAHA almost always finds the optimal local alignment. However, due to the use of various heuristics such as X-Dropoff and banding, this is not guaranteed.

### 2.5 Apply Optimal Query Coverage algorithm

Optionally, YAHA can report all alignments identified through the above steps. This feature is invaluable when it is important to gain knowledge about the uniqueness of a query sequence or the distribution of repeats in the reference genome. However, in order to define SV breakpoint locations, it is often preferable to ignore the potentially large numbers of irrelevant alignments that arise from repeats embedded within larger, more unique portions of the query. For this purpose we have devised an algorithm called OQC, which finds the set of alignments that cover the length of the query with the maximum coverage score. This Optimal Coverage Set (OCS) is composed of one or more ‘Primary Alignments’. This algorithm greatly aids in reconstructing breakpoint architecture and is a crucial, and novel, feature of YAHA.

To find the OCS, we use a max-path DAG algorithm similar to that described in Section 2.3 above. The nodes now represent the alignments, and the edges represent one alignment being included with another in the OCS (Fig. 1D). We again presort the alignments by starting QO to avoid creating the edges. In cases where two alignments overlap at the breakpoint, as occurs when structural variants are generated by homology dependent mechanisms, the score of the better alignment in the overlap region is used. In order to avoid an overly fractured OCS, a penalty is applied for each split between adjacent alignments. This penalty is the product of two factors. The first is a user supplied parameter called the Breakpoint Penalty (BP). The second is a Genomic Distance Penalty (GDP) calculated as log_10_ of the number of base pairs along the reference genome between the two alignments. The user can specify a maximum GDP (maxGDP). Alignments on separate chromosomes always incur the maxGDP. Through these two parameters, the user can control how sensitive the query coverage score is to genomic distance, and how large the non-overlapping portion of an alignment must be before it is included in the OCS. With a relatively high maxGDP, collections of alignments near each other on a reference chromosome will be favored, helping to identify deletions, tandem duplications and inversions. A low maxGDP will be more neutral to genomic distance. A higher BP will favor alignment sets with fewer, larger, alignments.

We believe that this OQC calculation allows for the discovery of biologically meaningful collections of alignments. We show below that YAHA’s OQC algorithm is better at discovering SV events than the heuristic approach used by BWA-SW for finding split alignments.

### 2.6 Apply Filter By Similarity algorithm

Optionally, we next perform the FBS step to identify ‘Secondary Alignments’ that have a high length overlap and score agreement with a primary alignment (Fig. 1D). This allows the user to gain knowledge of repetitive mappings specifically for those sections of the query that comprise a primary alignment. This is required for clustering algorithms designed to identify breakpoints in repetitive genomic regions, and may be useful for characterizing the repetitive structure of fully sequenced reference genomes (Bailey *et al.*, 2002) This novel feature of YAHA combines the utility of finding large numbers of alignments with the advantage of defining the optimal collection along the query.

## 3 RESULTS

To demonstrate YAHA’s power and flexibility, we measure its performance in three test scenarios. First, we show that YAHA is sufficiently sensitive to find large numbers of alignments for queries with repeated (sub)sequences. Second, we measure YAHA’s ability to accurately find alignments when using the OQC algorithm for non-chimeric queries. Third, we test the OQC and FBS algorithms by measuring YAHA’s ability to detect SV breakpoints in chimeric queries. For each test, we compare YAHA to what we believe to be best of breed among commonly used aligners for that specific task. In the sensitivity test, we compare against MegaBLAST because it is generally considered one of the most sensitive heuristic aligners for finding a large number of alignments in a practical amount of time. Because we use the same indexing strategy as SSAHA2, we also include it in this test. BWA-SW only reports primary alignments so cannot be included in the sensitivity comparison. We do not include MegaBLAST or SSAHA2 in the accuracy or SV detection tests because neither has any strategy for finding an OCS. For these tests, we compare our results to BWA-SW which is the most widely used long-read aligner and the most challenging competitor to YAHA for finding primary alignments on either chimeric or non-chimeric queries. In particular, it has already been shown that BWA-SW outperforms SSAHA2 and BLAT on non-chimeric reads (Li and Durbin, 2010). In all these tests, CPU time is an important metric, as any alignment task is easy to perform by brute force if an aligner is given unlimited computer resources. Finally, we note that it is difficult to compare results from different aligners because most are highly parameterizable, but do not share all the same parameters. We have made a considerable effort to select the most effective parameters to use for YAHA and the other aligners, but we cannot exclude the possibility that untested parameter combinations might produce superior results to those we present here.

The data for the accuracy test was generated using WGSIM (Li *et al.*, 2009) to sample reads from the hg18 reference genome with the lengths and error rates shown in Table 2. For the sensitivity test, we focus on the first of these datasets; 100 000 queries of length 100 with a 2% error rate. For the SV detection test, we used our own tool, SVsim, to simulate SV events of various types.

All tests were run on a server class machine with 4 Xeon X7350 processors, 128 GB of shared RAM, running CentOS 5.5. YAHA’s 15/1 index and compressed reference total 15.5 GB, SSAHA’s total 22.3 GB and BWA-SW’s index and reference total 7.4 GB. However, we believe that index size is a minor concern given modern computing environments.

### 3.1 Sensitivity test

To test sensitivity, we ran YAHA bypassing the OQC and FBS algorithms and output all alignments that pass applicable thresholds. An issue arises in trying to compare results from YAHA, MegaBLAST, and SSAHA2 because they do not have the same threshold parameters, and SSAHA2 does not support AGS. To equalize results, we applied an external filter to keep only alignments greater than or equal to 50 bp in length, and ran each aligner with its thresholds set so that such alignments were not filtered out internally. Also, each aligner uses different criteria to determine if two alignments are ‘distinct’ enough to report separately. To account for this, we filtered out alignments that are overlapping on the reference with another alignment for the same query. We refer to the final set of filtered alignments as ‘GE50U’ alignments. As there is no practical way to determine *every* location in the reference that can be aligned to a given query, we measure *relative* sensitivity in this test. Version 2.2.19 of MegaBLAST was run using parameter settings that are as sensitive as possible with a seed length of 15. The result is the baseline against which we compare the sensitivity of YAHA and SSAHA2.

Seven aligner runs are used in this test (Table 1). We study four YAHA runs with parameter settings representing different points along the sensitivity spectrum. For three of the runs, we used a minMatch of 15 (one seed hit) and a maxHits of 65 525 sampled (Y1), 10 000 sampled (Y2) and 10 000 unsampled (Y3). The fourth run (Y4) used faster but less sensitive parameters; maxHits of 650 unsampled and minMatch of 20. We also study two runs of version 2.5.1 of SSAHA2. The first (S1) used SSAHA2’s built-in 454 mode, which implies many SSAHA2 parameters, including ones for the Crossmatch back-end, and a 13/3 index. SSAHA2’s default and solexa modes do not perform well in this test and are not included. The second (S2) also used the 454 mode Crossmatch parameters, but with a 15/1 index, SSAHA2 default value of 10 000 for maxHits, and a minMatch of 1. These parameters make this run directly comparable to the Y3 run.

**Table 1:** Results of the sensitivity test

			Alignments				Versus MegaBLAST		
Run	Aligner and parameters	CPU secs	Total	GE50U	A/Sec	GE50U/Sec	>M	=M	<M
M	MegaBLAST: wordLen = 15, score = 15	190 773	4 012 294 854	1 827 862 215	21 032	9581	0	100 000	0
Y1	YAHA: minMatch = 15, maxHits = 65 525 S	160 501	6 085 988 010	2 343 744 189	37 919	14 603	30 638	68 357	1005
Y2	YAHA: minMatch = 15, maxHits = 10 000 S	91 097	3 403 790 544	1 470 115 221	37 364	16 138	23 789	68 387	7824
Y3	YAHA: minMatch = 15, maxHits = 10 000	22 385	950 852 793	327 644 121	42 477	14 637	20 021	68 371	11 608
Y4	YAHA: minMatch = 20, maxHits = 650	284	11 680 597	6 796 153	41 129	23 930	716	69 536	29 748
S1	SSAHA2: 454 mode	1850	6 066 013	5 488 465	3279	2967	834	66 634	32 532
S2	SSAHA2: minMatch = 1, maxHits = 10 000	937	2 633 833	1 101 352	2811	1175	120	65 622	34 258

The first two columns give the name and aligner parameters, column 3 gives the runtimes, columns 4–7 contain the total alignments, GE50U alignments, total alignments/second, and GE50U alignments/second, and the last three columns show the number of queries with >, =, or < the number of alignments as the MegaBLAST run.

Table 1 shows the test results. The percentage of queries with the same number of GE50U alignments as M is similar across runs. In fact, 64% of the queries produce the same number of GE50U alignments across all seven runs. Of these, 97.7% produce a single GE50U alignment. This indicates there is high agreement between aligners for queries that map to unique locations on the reference. In addition, all of the YAHA runs produce significantly more total and GE50U alignments per second than any other aligner runs.

**Fig. 2:**
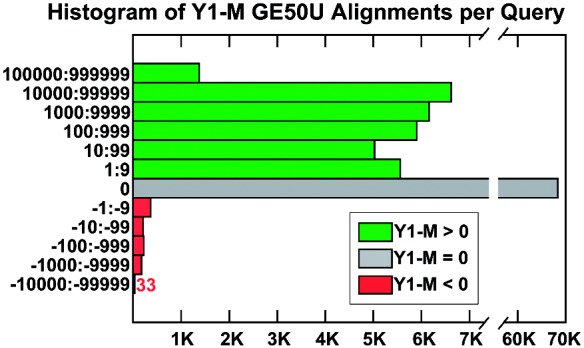
Histogram of the number of queries in the Y1 YAHA run with varying numbers of greater, equal and fewer GE50U alignments than MegaBLAST (M). Note the log_10_ scale bucket sizes. The total number of queries above 0 is 30 638 and below 0 is 1005 as in Table 1.

Y1 uses the most sensitive YAHA parameters possible for a 15/1 index, and produces more total alignments, more GE50U alignments and more queries with a greater number of GE50U alignments, in less runtime than MegaBLAST. This is a striking result. We analyze it further by expanding the last three columns of the Y1 row of Table 1 into a histogram of the difference in the number of GE50U alignments in the Y1 run versus M (Fig. 2). This shows that the two aligners can differ in the number of alignments for queries with highly repetitive (sub)sequences by five orders of magnitude. Yet, the graph is highly skewed in Y1’s favor, showing that YAHA is significantly more sensitive than MegaBLAST at identifying large numbers of alignments for such queries, while using less runtime.

YAHA greatly outperforms SSAHA2. For example, Y3 and S2 use comparable parameters, yet Y3 reports ~297× more GE50U alignments at ~12× greater speed (GE50U/s). The algorithmic basis for this dramatic difference in sensitivity is unclear. While S1 fared somewhat better, we note that this disparity persists across a wide range of SSAHA2 parameters (data not shown).

Y2 and Y3 agree in all parameter settings except Y2 uses an index with random sampling of *k*-mers that appear more than 10 000 times in the hg18 genome. More than 99.9996% of all 15-mers appear fewer than 10 000 times in hg18, yet Y2 requires ~4× the runtime and produces ~4.5× the number of GE50U alignments. This shows that the very few highly repetitive *k*-mers greatly impact queries that contain them. It also shows, together with the use of sampling in Y1, the improvement in sensitivity derived from sampling such *k*-mers instead of excluding them.

Over 99.99% of all possible 15-mers appear fewer than 650 times in hg18. Therefore, even without using sampling, this acts as a reasonable maxHits cutoff for relatively fast runs. Y4 uses this maxHits threshold, and further reduces runtimes by using a minMatch of 20 instead of 15. These prove to be effective settings, as Y4 produces ~1.6× as many GE50U alignments per CPU second as the other YAHA runs, and ~2.5× as many as M. Given these results, we use 650 as the maxHits threshold for YAHA in the accuracy test below. The nearly 11.7 million total alignments in the Y4 run act as the input to the OQC algorithm across the 100 000 queries in the first dataset of the accuracy test as we discuss next.

### 3.2 Accuracy test

We now compare the accuracy of YAHA to BWA-SW in finding primary alignments. We use the same process for generating synthetic queries as used in the accuracy test in the BWA-SW paper. However, we use slightly different accuracy metrics. In that study, they determined the false positive rate using ‘mapping quality’, a heuristically determined measure of an aligner’s confidence in the uniqueness of its alignments. Instead, we use as the benchmark the optimal alignment and score of each generated read at the source reference location found by SSEARCH, a tool from the FASTA suite (Pearson and Lipman, 1988) that uses full SW to find the best local alignment. For each aligner, we place each query into one of four categories. If no alignment was generated for a query, it is a false negative. If a primary alignment matches the optimal alignment found by SSEARCH, it is a ‘match’. For each remaining query, we independently calculated the best non-overlapping score of the alignment(s), called the Coverage Score (CS). If the CS is less than the SSEARCH score, it is a false positive. If the CS equals or exceeds the SSEARCH score, the alignment(s) produced are at least as viable as the one from the source location. Such queries are not real false positives, and are reported in their own category. We believe that the use of externally verified alignment scores is a far less biased and more precise metric of aligner accuracy because it isolates the effects of alignment heuristics from the mapping quality heuristics.

BWA-SW version 0.5.8 was run with default settings. YAHA was run with OQC turned on, BP = 5, maximum GDP (maxGDP) = 5, maxHits = 650, and varying values for minMatch of 20, 26, 38, 100 and 500 for the different QLs, respectively. Table 2 shows the results of the BWA-SW and YAHA runs using these 15 datasets. The aligners differ most on the 100-mer queries with 5% and 10% error rates, and the 200-mer queries with 10% error rate. These three datasets are the most challenging for both aligners, but YAHA has a significantly lower false positive and especially false negative rate, accounting for most of the large difference in these metrics shown in the Totals column. YAHA has a lower false negative rate for six of the datasets, versus five for BWA-SW. However, for three of the datasets in which BWA-SW has a lower false negative rate, YAHA merely fails to align a single query. YAHA has a lower false positive rate for eleven of the datasets, versus two for BWA-SW. YAHA has a lower sum of error rates for ten of the datasets, versus two for BWA-SW. The aggregation of results by query and by dataset both contain biases, albeit different ones. The former is biased by the fact that the datasets do not all contain the same number of queries, while the latter is biased by datasets with a small number of queries that differ. Nonetheless, YAHA achieves better results than BWA-SW for both aggregation strategies.

As a further test of accuracy, we compare all matching alignment scores against the optimal scores determined by SSEARCH. For reasons discussed in Section 2, YAHA produced sub-optimal scores for 20 of 522 244 matching alignments (0.0038%). BWA-SW produced a sub-optimal score for 1 of 483 786 matching alignments (0.0002%). All the sub-optimal alignments from both aligners were from datasets with a 10% error rate.

**Table 2:** Accuracy comparison of YAHA to BWA-SW over 15 datasets generated in a similar fashion as those in the BWA-SW paper

	100 K 100 bp Reads			50 K 200 bp Reads			20 K 500 bp Reads			10 K 1000 bp Reads			1 K 10 000 bp Reads			
Metric	2%	5%	10%	2%	5%	10%	2%	5%	10%	2%	5%	10%	2%	5%	10%	TOTALS
BWA-SW																
CPU secs	160	135	102	220	186	140	259	194	154	219	193	142	155	146	129	2534
% False negatives	0.44	5.21	27.4	0.00	0.13	5.44	0.00	0.00	0.10	0.00	0.00	0.00	0.00	0.00	0.00	6.61
% Matching	96.0	89.3	64.0	98.2	97.5	89.3	98.9	98.9	98.2	99.3	99.2	99.2	99.8	99.5	98.1	89.10
% CS ≥ SSEARCH	2.96	2.92	2.69	1.74	1.71	1.66	1.09	1.02	1.13	0.68	0.74	0.66	0.20	0.50	0.90	2.21
% False positives	0.56	2.53	5.85	0.11	0.70	3.63	0.01	0.12	0.56	0.02	0.02	0.12	0.00	0.00	1.00	2.09
% Total error	1.00	7.74	33.3	0.11	0.83	9.08	0.01	0.12	0.66	0.02	0.02	0.12	0.00	0.00	1.00	8.70
YAHA																
CPU Secs	284	241	176	212	171	109	245	188	112	108	86	58	81	79	66	2216
% False negatives	0.32	0.12	0.55	0.03	0.00	0.02	0.00	0.01	0.00	0.01	0.01	0.07	0.00	0.00	0.00	0.19
% Matching	96.3	95.5	91.8	98.1	97.9	97.2	99.0	98.8	98.8	99.3	99.2	99.1	99.9	99.7	99.2	96.18
% CS ≥ SSEARCH	2.83	3.03	3.72	1.71	1.77	1.87	1.04	1.18	1.06	0.71	0.81	0.76	0.10	0.30	0.80	2.42
% False positives	0.55	1.31	3.97	0.16	0.34	0.93	0.02	0.02	0.11	0.00	0.01	0.03	0.00	0.00	0.00	1.21
% Total error	0.87	1.43	4.52	0.19	0.35	0.96	0.02	0.03	0.11	0.01	0.02	0.10	0.00	0.00	0.00	1.40

The column headings indicate the read length and number of reads in each group, as well as the error rates impressed on the reads. Each query is put into one of four categories (row) depending on the accuracy of the alignment (see text for details). The CPU time in seconds, and total error rate for each run are also shown. The right-most column shows the aggregate runtimes and category percentages.

Summed over the datasets, YAHA uses less CPU time than BWA-SW. This is impressive given that YAHA considers many more alignments. For example, in Table 1 the Y4 run on 100-mers at 2% error rate produces ~11.7 million alignments, from which the OQC algorithm selects 99 696 primary alignments. In contrast, BWA-SW severely restricts the number of potential alignments early during query processing. By considering many more alignments, YAHA achieves greater accuracy. The advantages of using many alignments as input to OQC becomes more apparent in our test of SV breakpoint detection in the next section.

### 3.3 SV detection test

**Fig. 3:**
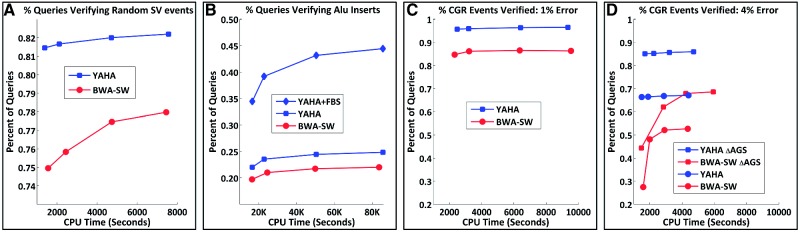
Shown are graphs of the percentage of queries with which each aligner correctly verified an SV breakpoint for various types of SV events versus the amount of CPU time consumed. Note the large improvement with the inclusion of YAHA’s secondary alignments in the Alu dataset. Also note the marked improvement for both BWA-SW and YAHA in the CGR dataset with 4% error rate by changing the AGS parameters to lower the penalty for indels relative to replacements. Still, YAHA outperforms BWA-SW with both sets of AGS parameters. Graphs C and D are shown with the same axes to ease comparison.

Finally, we compare YAHA to BWA-SW in their ability to correctly identify SV breakpoints with split-read mappings. This is an important criterion for evaluating long read aligners, because as read lengths grow, split-read mapping is rapidly replacing PEM as the method of choice for SV detection. We constructed three simulated datasets using SVsim, a tool we devised for this purpose (Faust and Hall, in preparation). First, we simulated 10 000 SV events with lengths from 100 to 10 K bases in random genome locations with equal numbers of deletions, tandem duplications, and inversions, as well as insertions from a random distant genome location. For events of length ≤500, we generated a single ‘contig’ spanning the event, with 500 flanking bases on each side. For larger events, we generated a contig for only the left breakpoint, with 500 flanking bases. We then generated 500-mer reads by sampling these contigs with WGSIM using a 2% error rate and 5× coverage. We examined only a single breakpoint for each variant, yielding 10 000 total breakpoint calls. BWA-SW was run with default settings except the *z* parameter was set to 1, 2, 5 and 10 to investigate the trade-off between runtime and sensitivity. YAHA was run with similar parameters as in the accuracy test, using a minMatch of 25 with increasing values of maxHits. We measured the percentage of queries with a split alignment that verified the correct SV breakpoint (within 5 bases) and the total number of verified breakpoints (Fig. 3A).

Both aligners perform very well on this dataset, identifying ~98% of the simulated breakpoints. However, YAHA verifies breakpoints in more queries than BWA-SW at comparable runtimes. This test shows that both aligners are quite effective at identifying isolated SV breakpoints in random (mostly non-repetitive) genomic regions.

To investigate performance at breakpoints involving repetitive sequences, we simulated 10 000 Alu insertion events. We randomly selected 1000 intact Alu elements with minimal divergence to the canonical active elements (milliDev ≤10 and length ≥300) from the UCSC RepeatMasker annotation track, and injected each into 10 random genome locations. Read simulations and performance metrics are as above. This is a challenging test, because to detect an Alu insertion by split-read mapping, the breakpoint-containing read must be aligned correctly not only to the flanking sequence at the ‘recipient’ locus (where the Alu inserted), but also to the Alu element at the correct ‘donor’ locus. This is difficult given the extremely large number of Alu elements in the reference genome, the high DNA sequence similarity shared between them, and the simulated error rate in the reads.

As a result, both aligners identify fewer breakpoints from far fewer queries for Alu insertions than for the standard SVs. Yet, YAHA again identifies slightly more breakpoints (not shown) from more queries (Fig. 3B). For example, using ~50 K CPU seconds YAHA identifies 79.6% of breakpoints from 24.5% of queries, while BWA-SW finds 76.8% of breakpoints from 21.7% of queries. In addition, this test shows the utility of YAHA’s FBS algorithm. When both primary and secondary alignments are taken into account, YAHA identifies 96.3% of breakpoints from 43.2% of queries. These additional alignments enable the discovery of repetitive element insertion events using combinatorial clustering algorithms (Hormozdiari *et al.*, 2009; Quinlan *et al.*, 2010).

Recent evidence indicates that complex genomic rearrangements (CGRs) are a common form of SV in both normal and cancer genomes (Quinlan and Hall, 2012). The most extreme example of this is chromothripsis (Stephens *et al.*, 2011), where chromosome regions are extensively rearranged due to the repair of chromosome shattering events involving hundreds of breakpoints. CGR events pose a unique challenge for breakpoint discovery because, with long-reads or assembled contigs, numerous breakpoints may be present on a single query. YAHA’s OQC algorithm is designed to select the optimal collection of alignments and should handle such situations better than heuristic strategies. To test this, we simulated 1500 chromosome shattering events each with a total length of ~30 kilobases. Of these, 1000 involve a single random genomic location, and 500 combine fragments from two different genomic locations. Fragments were generated from random locations within the selected regions, with an average size of 300 and a minimum size of 50. Of these fragments, 30% were deleted, 10% duplicated, and 50% inverted. The resulting collection of fragments was then randomly shuffled and ‘ligated’ into a single contig. This generated a total of 129 915 CGR breakpoints. The contigs were used directly as long reads after impressing two different error profiles. The first models a contig reconstructed via *de novo* assembly of short reads, and has a 1% error rate, 10% of which are indels. The second models a single long read from third generation sequencing technology, such as the forthcoming Oxford Nanopore instrument, and has a 4% error rate, 90% of which are indels.

YAHA greatly outperforms BWA-SW with the long CGR contigs. In the 1% error profile data, YAHA finds ~96% of the breakpoints, versus ~86% for BWA-SW (Fig. 3C). In the 4% error profile data, using the default AGS parameters, YAHA finds ~67% of the breakpoints regardless of the maxHits setting, while BWA-SW finds from 27.5% to 52.7% of the breakpoints as the *z* parameter is increased. BWA-SW does not perform well on this test with its default *z* = 1, and requires ~2× the runtime of YAHA to approach its sensitivity asymptote (Fig. 3D).

Both aligners use the same default AGS parameter settings (Match = +1, Mismatch = −3, GapOpen = −5, GapBase = −2). However, these parameter settings are tuned for low error rates and especially low indel rates, and are not optimal for the high-indel CGR dataset with a 4% error rate. Thus, we re-ran both aligners against the 4% error rate CGR dataset with AGS parameters that increase the relative penalty for replacements versus indels (Mismatch = −5, GapOpen = −2, GapBase = −1). While both aligners now do significantly better, YAHA still far outperforms BWA-SW (Fig. 3D). BWA-SW now finds between 44% and 69% of the breakpoints, while YAHA finds ~85%. This shows the importance of using an alignment scoring strategy, such as parameterized AGS, to handle the high error/indel rates that exist in current and future third generation sequencing technologies (Schadt *et al.*, 2010).

The inclusion of a genomic distance penalty in the objective function of YAHA’s OQC algorithm undoubtedly aids its performance in these tests, as it allows YAHA to favor collections of alignments for the OCS that are near each other in the genome.

## 4 CONCLUSION

We have shown that YAHA is a fast and effective all-purpose aligner that outperforms best-in-class tools for very three different tasks: (i) reporting all mappings per query; (ii) reporting the single best mapping and (iii) identifying split-mappings that define one or more SV breakpoints within a query. YAHA’s main strength as a general alignment tool is that it simply attempts to identify all possible matches according to the parameters set by the user. YAHA is able to explore many possible alignments without sacrificing speed through the use of a number of pre-existing and novel heuristics, as well as optimized implementations of computationally intensive procedures such as seed-match sorting, banded SW and max-path graph algorithms.

The most important and novel feature of YAHA is that it determines the set of the alignments that cover a query using an algorithm that provably optimizes a biologically relevant objective function tuned to SV breakpoint detection. This capability, as well as the ability to report secondary alignments using FBS, will be invaluable for SV mapping experiments that rely on long reads or assembled contigs. As we have shown, these methods are especially powerful for defining breakpoints caused by repetitive elements, and for reconstructing highly complex genome rearrangements.
